# Cardiac Stem Cell Secretome Protects Cardiomyocytes from Hypoxic Injury Partly via Monocyte Chemotactic Protein-1-Dependent Mechanism

**DOI:** 10.3390/ijms17060800

**Published:** 2016-05-24

**Authors:** Chi-Yeon Park, Seung-Cheol Choi, Jong-Ho Kim, Ji-Hyun Choi, Hyung Joon Joo, Soon Jun Hong, Do-Sun Lim

**Affiliations:** 1 Department of Cardiology, Cardiovascular Center, College of Medicine, Korea University, Seoul 02841, Korea; chiyoun@korea.ac.kr (C.-Y.P.); choisc86@gmail.com (S.-C.C.); mecey@naver.com (J.-H.K.); j2610@hanmail.net (J.-H.C.); drjoohj@gmail.com (H.J.J.); psyche94@gmail.com (S.J.H.); 2 Department of Cardiology, Cardiovascular Center, Korea University Anam Hospital, 73, Inchon-ro, Seongbuk-gu, Seoul 02841, Korea

**Keywords:** cardiac stem cells, immortalization, secretome, MCP-1, cardiomyocyte survival

## Abstract

Cardiac stem cells (CSCs) were known to secrete diverse paracrine factors leading to functional improvement and beneficial left ventricular remodeling via activation of the endogenous pro-survival signaling pathway. However, little is known about the paracrine factors secreted by CSCs and their roles in cardiomyocyte survival during hypoxic condition mimicking the post-myocardial infarction environment. We established Sca-1+/CD31− human telomerase reverse transcriptase-immortalized CSCs (Sca-1+/CD31− CSCs*^hTERT^*), evaluated their stem cell properties, and paracrine potential in cardiomyocyte survival during hypoxia-induced injury. Sca-1+/CD31− CSCs*^hTERT^* sustained proliferation ability even after long-term culture exceeding 100 population doublings, and represented multi-differentiation potential into cardiomyogenic, endothelial, adipogenic, and osteogenic lineages. Dominant factors secreted from Sca-1+/CD31− CSCs*^hTERT^* were EGF, TGF-β1, IGF-1, IGF-2, MCP-1, HGF R, and IL-6. Among these, MCP-1 was the most predominant factor in Sca-1+/CD31− CSCs*^hTERT^* conditioned medium (CM). Sca-1+/CD31− CSCs*^hTERT^* CM increased survival and reduced apoptosis of HL-1 cardiomyocytes during hypoxic injury. MCP-1 silencing in Sca-1+/CD31− CSCs*^hTERT^* CM resulted in a significant reduction in cardiomyocyte apoptosis. We demonstrated that Sca-1+/CD31− CSCs*^hTERT^* exhibited long-term proliferation capacity and multi-differentiation potential. Sca-1+/CD31− CSCs*^hTERT^* CM protected cardiomyocytes from hypoxic injury partly via MCP-1-dependent mechanism. Thus, they are valuable sources for *in vitro* and *in vivo* studies in the cardiovascular field.

## 1. Introduction

Cardiac Sca-1+ cells showed cardiac stem cell (CSC) properties differentiating into cardiac and endothelial cells, as evidenced by *in vitro* and *in vivo* studies [1,2,3,4,5]. Knockdown of Sca-1 transcripts in CSCs resulted in significant inhibition of proliferation and survival through Akt [6]. Sca-1+ CSCs were significantly increased in the mouse heart seven days after acute myocardial infarction (AMI) [7,8], and they migrated from a niche to the infarct zone to repair damaged myocytes after myocardial infarction (MI) under hypoxic conditions [9]. Sca-1 knockout revealed cardiac defects in myocardial contractility and repair consistent with impaired resident CSC proliferative capacity [1,10]. A significant and lasting contribution of Sca-1-derived cells to cardiomyocytes during normal aging were found [11]. Collectively, previous studies have demonstrated that Sca-1+ CSCs are valuable sources for myocardial renewal in the pathophysiological process as well as in the aging process of murine adult hearts. However, Sca-1+ CSCs were found to represent only 2% of total heart cells [1]. Therefore, small numbers of Sca-1+ CSCs present in the adult murine heart and their limited proliferative potential during *in vitro* culture restrict their use for *in vitro* and *in vivo* studies. 

Telomerase reverse transcriptase (*TERT*), a catalytic subunit of telomerase, serves a critical role in stem cell function and tissue homeostasis [12]. Several studies have demonstrated that primary stem cells inserted with the *TERT* gene have maintained long-term stemness *in vitro* and have been immortalized without chromosomal aberrations or characteristics of malignant transformation [13,14,15]. Recently, we also demonstrated that *TERT*-immortalized Sca-1+ adipose stem cells (ASCs*^TERT^*) exhibit stem cell properties similar to those of primary ASCs [16]. Interestingly, *TERT*-expressing cells in adult hearts were associated with Sca-1 expression [6], indicating that Sca-1 is a valuable surface marker for CSCs exhibiting high *TERT* activity.

A number of studies have reported that stem cells secrete diverse cytokines, chemokines, and angiogenic and cardiogenic growth factors, resulting in improvement of cardiac function via activation of the endogenous signaling pathways [17,18]. We, and others [16,19,20,21] have demonstrated that functional improvement and beneficial left ventricular (LV) remodeling by stem cell transplantation into animal models of AMI have been primarily achieved through paracrine actions rather than direct transdifferentiation of the transplanted cells. However, little is known about paracrine factors secreted by CSC and their roles in cardiomyocyte survival during hypoxic condition *in vitro* mimicking the post-infarcted myocardial microenvironment.

The aims of this study were to establish *TERT*-immortalized Sca-1+ CSCs that exhibit stem cell properties similar to those of primary Sca-1+ CSCs, to analyze paracrine factors secreted by the immortalized CSCs, and to elucidate their role on cardiomyocyte survival during hypoxia-induced injury.

## 2. Results

### 2.1. Isolation of Sca-1+ CSCs from Adult Myocardium

**Figure 1:**
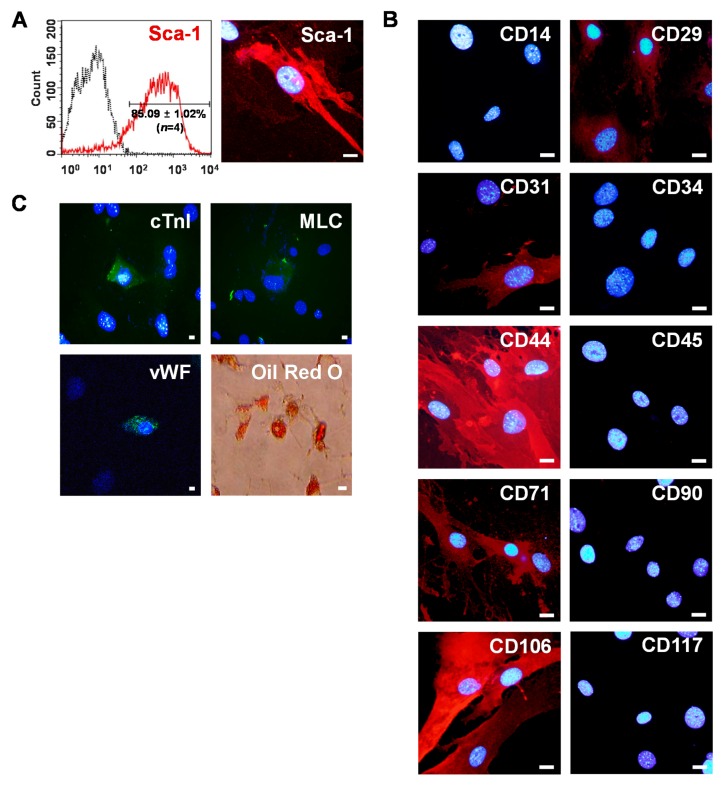
Isolation of mouse Sca-1+ CSCs from adult heart. (**A**) Sca-1+ CSCs were enriched by MACS with PE-conjugated anti-Sca-1 antibody and anti-PE micro beads. After sorting four rounds, ~86% of the cells expressed Sca-1 as determined by flow cytometry (**left**). CSCs expressing intense Sca-1 signals were observed under confocal microscopy after immunostaining with anti-Sca-1 antibodies (**right**). Scale bars = 20 μm; (**B**) characterization of Sca-1+ CSCs. Sca-1+ CSCs were stained with anti-CD14, -CD29, -CD31, -CD34, -CD44, -CD45, -CD71, -CD90, -CD106, and CD117 antibodies and visualized with Alexa Fluor 594 secondary antibodies (red). Scale bars = 20 μm; and (**C**) differentiation potential of Sca-1+ CSCs. Cardiac, endothelial, and adipogenic differentiation were confirmed by immunostaining with cardiomyocyte markers (cTnI, MLC, green), an endothelial marker (vWF, green), and Oil-Red O staining (red), respectively. Nuclei were stained with DAPI (blue). Scale bars = 20 μm.

Primary Sca-1+ CSCs were isolated after collagenase treatment followed by magnetic activated cell sorting (MACS) from mouse adult myocardium. Flow cytometry showed that Sca-1+ CSCs were more than 86% pure (Figure 1A). In confocal microscopic analysis, intense Sca-1 signals were shown in MACS-purified Sca-1+ CSCs with a fibroblast-like morphology (Figure 1A). For phenotypic characterization of Sca-1+ CSCs, cells were immunostained with antibodies to stem cells or cell lineage markers. Sca-1+ CSCs strongly expressed CD44 and CD106 (Figure 1B). They also moderately expressed CD29 and CD71. Interestingly, primary Sca-1+ CSCs were heterogeneous with respect to CD31 expression. Some primary Sca-1+ CSCs were negative for CD31 but others were positive for CD31. In accordance with our observation, previous studies [3,5,6,7,8] also reported that cardiac Sca-1+/CD31− cells exhibit multipotent differentiation potential *in vitro* and their therapeutic potential in experimental myocardial infarction models, whereas cardiac Sca-1+/CD31+ cells showed endothelial-like characteristics.

The multi-potency of primary Sca-1+ CSCs was investigated by their ability to differentiate into cardiac, endothelial, and adipogenic lineages. Sca-1+ CSCs were differentiated into cardiomyocytes expressing cardiac troponin I (cTnI) and myosin light chain (MLC) after treatment with 1 µM 5-azacytidine for 21 days (Figure 1C). Immunofluorescence staining showed that Sca-1+ CSCs differentiated into endothelial cells that express an endothelial cell specific marker von Willebrand factor (vWF) after being induced by 20 ng/mL vascular endothelial growth factor (VEGF) for 21 days (Figure 1C). Adipocytes showing Oil red O-positive staining of large lipid vacuoles were generated by differentiating Sca-1+ CSCs for 10 days in adipogenic differentiation medium. (Figure 1C).

### 2.2. Establishment of Human TERT (hTERT)-Immortalized Sca-1+ CSC Lines

**Figure 2:**
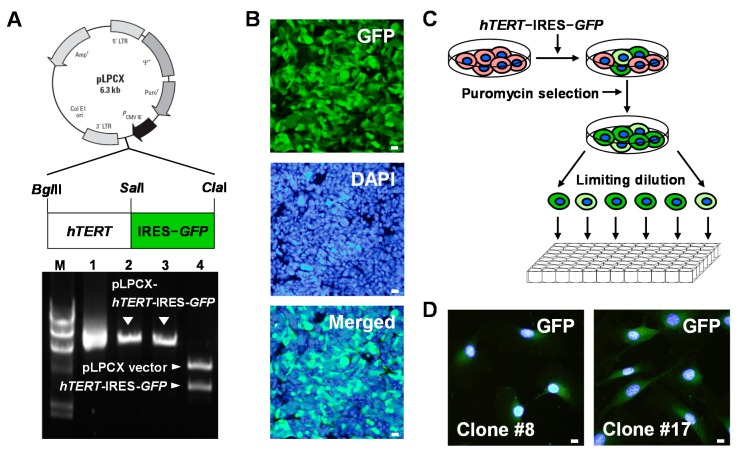
Establishment of *hTERT*-immortalized Sca-1+ CSC lines. (**A**) Schematic structure of pLPCX-*hTERT*-IRES-*GFP* (**top**). Correct construction of pLPCX-*hTERT*-IRES-*GFP* was confirmed by digestion with restriction enzymes (**bottom**), *Bgl*II (Lane 2), *Cla*I (Lane 3), or *Bgl*II*/Cla*I (Lane 4). Lane M: λ/*Hind*III marker. Lane 1: Supercoiled pLPCX-*hTER*T-IRES-*GFP*; (**B**) retroviruses were produced in 293GPG packaging cells by transfection with a retroviral vector encoding *hTERT*-IRES-*GFP* using Lipofectamine 2000. At 48 h post transfection, expression of GFP (green), a reporter gene was monitored in 293GPG cells. Scale bars = 20 μm; (**C**) Sca-1+ CSCs transduced with retroviruses expressing *hTERT*-IRES-*GFP* (green) were selected in 96-well plates at a single cell level by limiting dilution for 12 days; and (**D**) two putative Sca-1+ CSC lines were finally selected. Nuclei were stained with DAPI (blue).

To establish *hTERT*-immortalized mouse Sca-1+ CSC lines, a retroviral vector carrying *hTERT*-internal ribosome entry site (IRES)-green fluorescent protein (*GFP*) was constructed (Figure 2A). Retroviruses were produced in 293GPG packaging cells by transfection with a retroviral vector carrying *hTERT*-IRES-*GFP* (Figure 2B). MACS-purified Sca-1+ CSCs isolated from adult heart tissue were infected with retroviruses harboring *hTERT*-IRES-*GFP*, and then selected in a 10-cm culture dish containing puromycin during a three-week subculture period. Then, *hTERT*-immortalized Sca-1+ CSCs were further selected at a single cell level by limiting dilution in 96-well cell culture plates based on their GFP expression, morphology, and stem cell marker expression (Figure 2C and Table S1). Two putative CSC clones (Clones #8 and #17) were finally selected (Figure 2D and Table S1).

### 2.3. Evaluation of Stem Cell Potency of hTERT-Immortalized Sca-1+ CSC Lines

**Figure 3:**
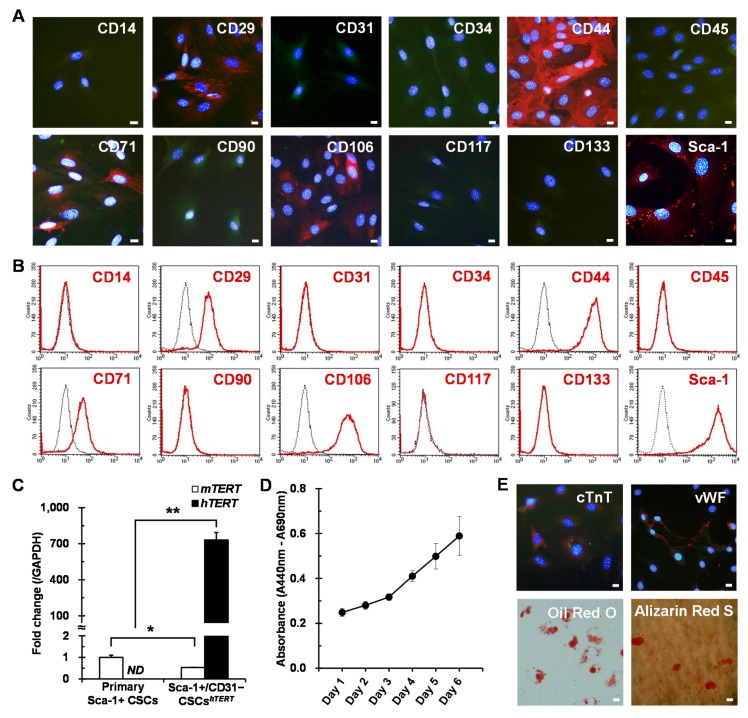
Sca-1+/CD31− CSCs*^hTERT^* exhibit stem cell potency. For phenotypic characterization, Sca-1+/CD31− CSCs*^hTERT^* expressing GFP (green) were analyzed by immunostaining (**A**) and flow cytometry (**B**) with different cell surface antibodies (red); (**C**) real-time PCR was performed to detect both endogenous *mTERT* and transduced *hTERT* transcripts in primary Sca-1+ CSCs and immortalized Sca-1+/CD31− CSCs*^hTERT^*. Data represent mean ± SD from three independent experiments (* *p* < 0.05; ** *p* < 0.01). *ND*, not determined; (**D**) for proliferation analysis, Sca-1+/CD31− CSCs*^hTERT^* were seeded at 1 × 10^3^ cells/well in 96-well microplates, cultured with Mesencult Basal Medium supplemented with cytokines for six days, and analyzed by WST-1 assay. Data represent mean ± SD from four independent experiments; and (**E**) Sca-1+/CD31− CSCs*^hTERT^* exhibit multi-differentiation potential. Differentiation was analyzed by immunostaining with a cardiomyocyte marker (cTnT, red), an endothelial marker (vWF, red), and by Oil-Red O staining (red) or Alizarin Red S staining (red). Nuclei were stained with DAPI (blue). Scale bars = 20 μm.

Phenotypic characterization of finally selected two *hTERT*-immortalized Sca-1+ CSC lines was further evaluated by immunostaining and flow cytometry with different cell surface antibodies. Two CSC lines were positive for CD29, CD44, CD71, and CD106 (Figure 3A,B and Figure S1A,B), showing phenotypic characteristics similar to those of primary Sca-1+ CSCs. Interestingly, one clone (Clone #8) was negative for CD31 (Figure 3A,B), but the other was positive for CD31 (Figure S1A,B). The two clones were designated as Sca-1+/CD31− CSCs*^hTERT^* and Sca-1+/CD31+ CSCs*^hTERT^*, respectively. Given the different phenotypic characteristics of the two cell lines, we selected Sca-1+/CD31− CSCs*^hTERT^* for further studies because Sca-1+/CD31− cells, but not Sca-1+/CD31+ cells were shown to possess more potent CSC properties [3,7,8].

Sca-1+/CD31− CSCs*^hTERT^* were cultured for about four months, corresponding to around 100 population doublings (PDs). Transduced *hTERT* transcripts were strongly expressed in Sca-1+/CD31− CSCs*^hTERT^*, whereas endogenous mouse *TERT* (*mTERT*) expression levels were significantly downregulated in Sca-1+/CD31− CSCs*^hTERT^* compared to primary Sca-1+ CSCs, possibly due to long-term culture exceeding ~100 PDs (Figure 3C). This result indicates that the stemness of the *hTERT*-immortalized CSC line is sustained by ectopic *hTERT* activity but not by endogenous *mTERT* activity. The PD time of Sca-1+/CD31− CSCs*^hTERT^* was approximately 32 h. Sca-1+/CD31− CSCs*^hTERT^* represent sustained proliferation ability even after long-term culture exceeding 100 PDs, as evaluated by WST-1 assay (Figure 3D). Sca-1+/CD31− CSCs*^hTERT^* represented multi-differentiation potential into cardiomyogenic, endothelial, adipogenic, and osteogenic lineages (Figure 3E), indicating that they have the capacity to differentiate into various mesodermal cell lineages.

### 2.4. Sca-1+/CD31− CSCs^hTERT^ Conditioned Medium (CM) Protects HL-1 Cardiomyocytes from Cobalt Chloride (CoCl_2_)-Induced Hypoxic Injury

**Figure 4:**
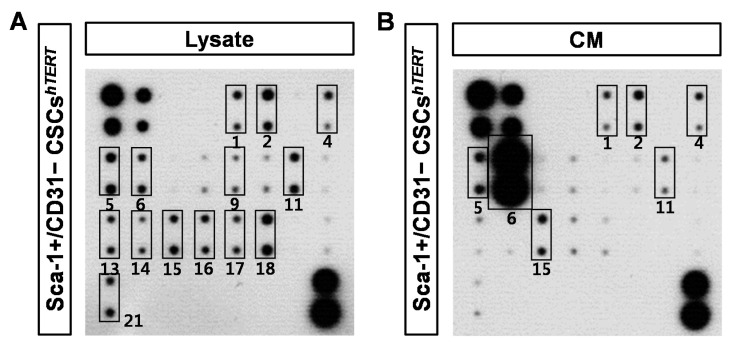

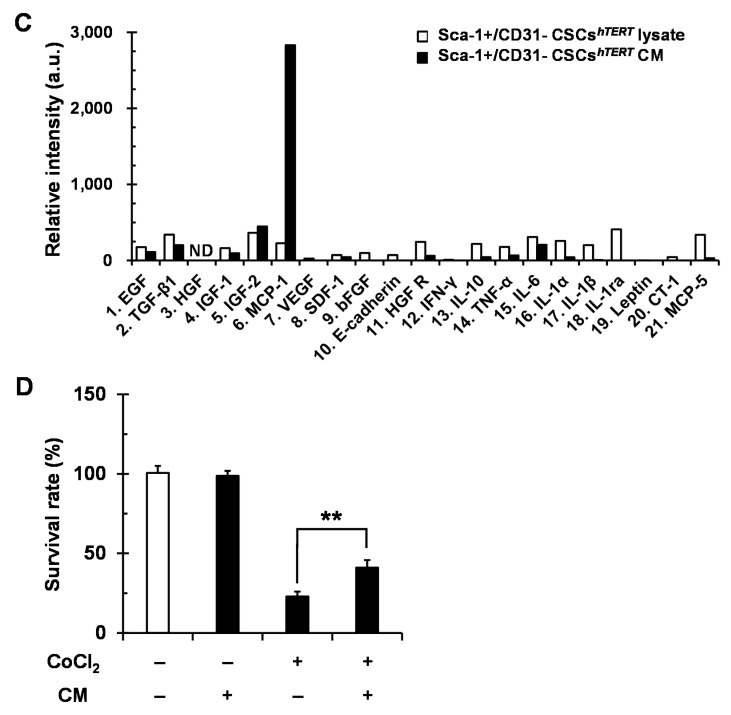
Sca-1+/CD31− CSCs*^hTERT^* CM protects HL-1 cardiomyocytes from CoCl_2_-induced hypoxic injury. Sca-1+/CD31− CSC*^hTERT^* lysate (**A**) and Sca-1+/CD31− CSCs*^hTERT^* CM (**B**) were subjected to a mouse cytokine antibody array detecting 21 cytokines in duplicate. 1. EGF; 2. TGF-β1; 3. HGF; 4. IGF-1; 5. IGF-2; 6. MCP-1; 7. VEGF; 8. SDF-1; 9. bFGF; 10. E-Cadherin; 11. HGF R; 12. IFN-γ; 13. IL-10; 14. TNF-α; 15. IL-6; 16. IL-1α; 17. IL-1β; 18. IL-1ra; 19. Leptin; 20. CT-1; and 21. MCP-5. Solid-lined boxes indicate dominant paracrine factors expressed in Sca-1+/CD31− CSCs*^hTERT^* lysate or Sca-1+/CD31− CSCs*^hTERT^* CM; (**C**) relative quantification of cytokine levels expressed in Sca-1+/CD31− CSCs*^hTERT^* lysate and Sca-1+/CD31− CSCs*^hTERT^* CM; and (**D**) viable cells were counted using a hemocytometer after staining with 0.2% trypan blue Sca-1+/CD31− CSCs*^hTERT^* CM in HL-1 cardiomyocytes treated with or without 150 μM CoCl_2_ for 24 h. Data represent mean ± SD from three independent experiments (** *p* < 0.01).

To elucidate which paracrine factors are secreted from Sca-1+/CD31− CSCs*^hTERT^*, a total of 21 growth factors and inflammatory cytokines from Sca-1+/CD31− CSCs*^hTERT^* lysate and CM were analyzed using mouse cytokine antibody arrays. Epidermal growth factor (EGF), transforming growth factor-beta1 (TGF-β1), insulin-like growth factor 1 (IGF-1), IGF-2, monocyte chemotactic protein-1 (MCP-1), basic fibroblast growth factor (bFGF), E-Cadherin, hepatocyte growth factor receptor (HGF R), interleukin-10 (IL-10), tumor necrosis factor-alpha (TNF-α), IL-6, IL-1α, IL-1β, IL-1ra, and MCP-5 were dominant factors in Sca-1+/CD31− CSCs*^hTERT^* lysate (Figure 4A). The dominant cytokines and chemokines secreted from Sca-1+/CD31− CSCs*^hTERT^* were EGF, TGF-β1, IGF-1, IGF-2, MCP-1, HGF R, and IL-6 (Figure 4B). Among these, MCP-1 was the most predominant factor in Sca-1+/CD31− CSCs*^hTERT^* CM (Figure 4B). Other inflammatory cytokines, interferon-gamma (IFN-γ), IL-10, TNF-α, IL-1α, IL-1β, IL-1ra, Leptin, cardiotrophin-1 (CT-1), and MCP-5, were very weakly detected in Sca-1+/CD31− CSCs*^hTERT^* CM (Figure 4B). Relative levels of 21 different cytokines and chemokines expressed in Sca-1+/CD31− CSCs*^hTERT^* lysate, and Sca-1+/CD31− CSCs*^hTERT^* CM were quantified by densitometry (Figure 4C). We next compared the paracrine factors secreted from primary Sca-1+ CSCs and Sca-1+/CD31− CSCs*^hTERT^*. MCP-1 was the most predominant factor secreted from both primary Sca-1+ CSCs and Sca-1+/CD31− CSCs*^hTERT^* (Figure S2A,B). Interestingly, we found that VEGF and IL-6 were severely reduced in Sca-1+/CD31− CSCs*^hTERT^* CM compared to primary Sca-1+ CSCs CM (Figure S2A–C).

CoCl_2_ treatment induced apoptotic death of cardiomyocytes through hypoxia-inducible factor-1-alpha (HIF-1α)-dependent stabilization of p53 protein [22,23,24]. Therefore, CoCl_2_ was used as a hypoxia mimicking reagent to investigate whether paracrine factors secreted from Sca-1+/CD31− CSCs*^hTERT^* protect from CoCl_2_-induced death of cardiomyocytes. Survival rate of HL-1 cardiomyocytes was significantly reduced by 22% by treatment with 150 μM CoCl_2_ for 24 h (Figure 4D) as determined by 0.2% trypan staining. However, we found that survival rate of CoCl_2_-treated HL-1 cardiomyocytes was significantly increased by 41% by incubation with Sca-1+/CD31− CSCs*^hTERT^* CM, indicating that Sca-1+/CD31− CSCs*^hTERT^* CM could protect HL-1 cardiomyocytes from CoCl_2_-induced cell death (Figure 4D).

### 2.5. Sca-1+/CD31− CSCs^hTERT^ CM Reduces Hypoxia-Induced Cardiomyocyte Apoptosis Partly via MCP-1-Dependent Mechanism

**Figure 5:**
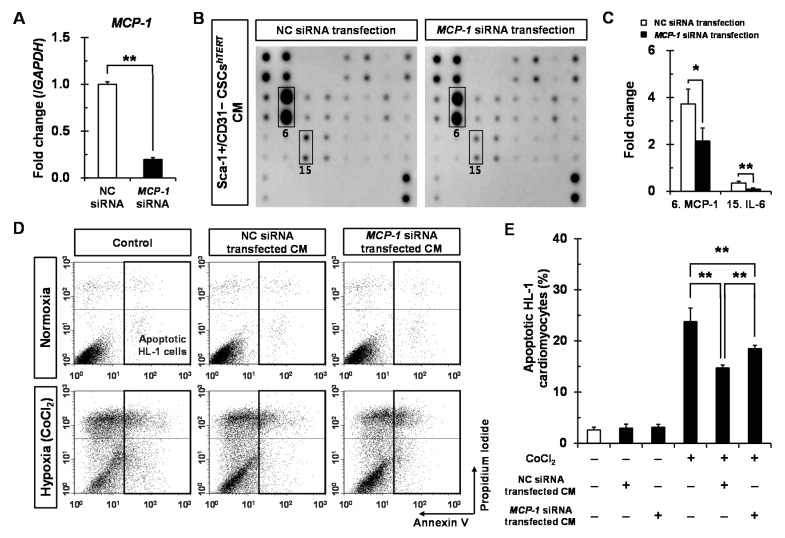

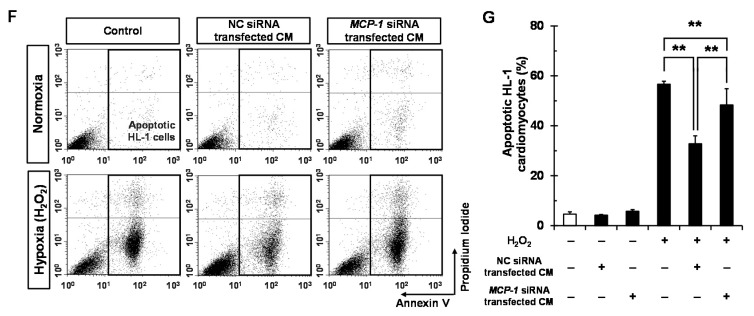
Sca-1+/CD31− CSCs*^hTERT^* CM protects HL-1 cardiomyocytes from hypoxic injury partly via MCP-1-dependent mechanism. Sca-1+/CD31− CSCs*^hTERT^* were cultured in six-well plates at a density of 5 × 10^4^ cells/well and transfected with 50 nM of *MCP-1* siRNA duplexes or NC siRNA duplexes using Lipofectamine RNAiMAX. After a 48 h transfection, *MCP-1* mRNA expression was assessed by real-time PCR (**A**); The average value of *MCP-1* mRNA was normalized to that of *GAPDH* for each sample. The data represent the mean ± SD from three experiments. Sca-1+/CD31− CSCs*^hTERT^* CM (**B**) after transfection of NC siRNA or *MCP-1* siRNA were subjected to densitometry and are presented as fold changes for MCP-1 (indicated by number 6) and IL-6 (indicated by number 15) (**C**), taking MCP-1 and IL-6 levels in NC siRNA-transfected Sca-1+/CD31− CSCs*^hTERT^* as a one-fold value, each in triplicate, * *p* < 0.05; ** *p* < 0.01 *vs.* NC siRNA; (**D**) representative flow cytometric analysis showing apoptotic effects on HL-1 cardiomyocytes treated with or without 150 μM CoCl_2_ for 24 h in the absence or presence of Sca-1+/CD31− CSCs*^hTERT^* CM transfected with NC siRNA or *MCP-1* siRNA. Bar graph of three independent experiments showing percentages of apoptotic HL-1 cardiomyocytes (**E**) treated with or without 150 μM CoCl_2_ for 24 h in the absence or presence of Sca-1+/CD31− CSCs*^hTERT^* CM transfected with NC siRNA or *MCP-1* siRNA (** *p* < 0.01); and (**F**) representative flow cytometric analysis showing apoptotic effects on HL-1 cardiomyocytes treated with or without 200 μM H_2_O_2_ for 24 h in the absence or presence of Sca-1+/CD31− CSCs*^hTERT^* CM transfected with NC siRNA or *MCP-1* siRNA; Bar graph of three independent experiments showing percentages of apoptotic HL-1 cardiomyocytes (**G**) treated with or without 200 μM H_2_O_2_ for 24 h in the absence or presence of Sca-1+/CD31− CSCs*^hTERT^* CM transfected with NC siRNA or *MCP-1* siRNA (** *p* < 0.01).

To define which paracrine factors secreted from Sca-1+/CD31− CSCs*^hTERT^* are responsible for protecting HL-1 cardiomyocytes from hypoxic cell death, we chose MCP-1 as a candidate among 21 growth factors and inflammatory cytokines because it was the most dominant factor secreted by Sca-1+/CD31− CSCs*^hTERT^*. We generated siRNA duplex against *MCP-1* to inhibit endogenous *MCP-1* expression in Sca-1+/CD31− CSCs*^hTERT^*. Real-time polymerase chain reaction (PCR) analysis showed that the silencing efficiency in *MCP-1* siRNA-transfected Sca-1+/CD31− CSCs*^hTERT^* was approximately 80% after a 48 h transfection (Figure 5A). To further elucidate which paracrine factors are affected in *MCP-1* siRNA-transfected Sca-1+/CD31− CSCs*^hTERT^*, a total of 21 growth factors and inflammatory cytokines from Sca-1+/CD31− CSCs*^hTERT^* CM after transfection of negative control (NC) siRNA or *MCP-1* siRNA were analyzed using mouse cytokine antibody arrays, and were subjected to densitometry. Expectedly, we observed that MCP-1 protein expression was significantly reduced by 57% in Sca-1+/CD31− CSCs*^hTERT^* CM by *MCP-1* siRNA transfection compared to that of the NC siRNA (Figure 5B,C). Interestingly, we found that IL-6 protein expression was also significantly reduced by 20% in *MCP-1* siRNA-transfected Sca-1+/CD31− CSCs*^hTERT^* CM compared to that of the NC siRNA after a 48 h transfection (Figure 5B,C).

We next examined the effect of *MCP-1* silencing in Sca-1+/CD31− CSCs*^hTERT^* on cardiomyocyte apoptosis. We found that NC siRNA-transfected Sca-1+/CD31− CSCs*^hTERT^* CM significantly reduced by 61% the proportion of apoptotic HL-1 cardiomyocytes during CoCl_2_-induced hypoxic injury compared to only CoCl_2_-treated control group (Figure 5D,E). *MCP-1* siRNA-transfected Sca-1+/CD31− CSCs*^hTERT^* CM resulted in a significant increase again (78%) in the proportion of apoptotic HL-1 cardiomyocytes compared to NC siRNA-transfected Sca-1+/CD31− CSCs*^hTERT^* CM (Figure 5D,E). Hydrogen peroxide (H_2_O_2_) induced oxidative stress-induced cardiomyocyte injuries [25,26]. Therefore, we also investigated the protective effects of Sca-1+/CD31− CSCs*^hTERT^* CM on H_2_O_2_-induced cardiomyocyte apoptosis. NC siRNA-transfected Sca-1+/CD31− CSCs*^hTERT^* CM significantly reduced by 58% the proportion of apoptotic HL-1 cardiomyocytes during H_2_O_2_-induced oxidative stress compared to only H_2_O_2_-treated control group (Figure 5F,G). *MCP-1* siRNA-transfected Sca-1+/CD31− CSCs*^hTERT^* CM resulted in a significant increase again (85%) in the proportion of apoptotic HL-1 cardiomyocytes compared to NC siRNA-transfected Sca-1+/CD31− CSCs*^hTERT^* CM (Figure 5F,G). These results indicate that MCP-1 plays an important role in protecting cardiomyocytes from hypoxia-induced apoptosis.

## 3. Discussion

The *hTERT* gene was used to immortalize Sca-1+ CSCs to minimize the occurrence of genetic and phenotypic instabilities caused by viral oncogenes, such as the simian virus 40 T antigen, adenoviral *E1A/E1B* genes, and human papillomavirus 16 *E6/E7* genes [13]. A retroviral vector encoding *hTERT*-IRES-*GFP* was constructed to direct gene expression from the immediate early promoter of cytomegalovirus and GFP expression through the IRES to prevent adverse effects on TERT activity due to the *GFP* sequence. In this study, we found that overexpression of ectopic *hTERT* was sufficient to immortalize mouse adult CSCs sustaining similar phenotypic characteristics and multi-differentiation potential more than 100 PDs to primary CSCs. Sca-1+/CD31− CSCs*^hTERT^* still exhibited strong GFP activity even after long-term *in vitro* culture exceeding ~100 PDs, indicating that these are very useful for monitoring cell movement or location.

A number of studies have also demonstrated that various adult stem cells inserted with the *TERT* gene have maintained long-term stemness *in vitro* and have been immortalized [13,14,15,27,28,29]. Freire *et al.* [30] also showed that Sca-1+ CSCs immortalized by the *mTERT* gene showed robust self-renewal capacity while preserving a stable phenotype in long-term culture using a similar approach to ours. Recently, we also established *hTERT*-immortalized mouse ASC^TERT^ [16], demonstrating the efficacy of *TERT* gene-mediated immortalization. In contrast, several studies have reported that *hTERT* alone was not sufficient for immortalization of human ASCs [31] or CD34+ human cord blood cells [32], revealing the necessity of co-transduction of viral genes with the *TERT* gene. These results suggest that immortalization of primary cells via overexpression of *TERT* alone could be cell type-dependent.

In this study, we found that MACS-purified Sca-1+ CSCs contain both CD31− and CD31+ subsets. Previous studies [7,8] have reported that Sca-1+ CSCs did express the typical mesenchymal stem-cell surface antigens CD29, CD44, CD73, and CD106 but not express the hematopoietic stem cell markers CD34, CD45, and the endothelial marker CD31 [6,33,34]. It has been shown that Sca-1+/CD31− cells but not Sca-1+/CD31+ cells represent a distinct cardiac progenitor cell population, capable of cardiomyogenic differentiation into mature cardiomyocytes [35]. Furthermore, therapeutic potentials of cardiac Sca-1+/CD31− mesenchymal stem-like cells in repair of injured heart have been demonstrated in experimental MI models [3,4,35]. Therefore, based on our findings and previous reports, we selected Sca-1+/CD31− CSCs*^hTERT^* for further studies because Sca-1+/CD31+ CSCs*^hTERT^* could be partially differentiated toward the endothelial cell lineage rather than resident CSCs.

In the current study, we investigated the effect of *MCP-1* silencing in Sca-1+/CD31− CSCs*^hTERT^* CM on CoCl_2_- or H_2_O_2_-induced cardiomyocyte apoptosis because MCP-1 was the most dominant factor in Sca-1+/CD31− CSCs*^hTERT^* CM. Although we significantly reduced both *MCP-1* mRNA and protein expression levels by *MCP-1* siRNA-transfection in Sca-1+/CD31− CSCs*^hTERT^*, but a large amount of MCP-1 was still secreted from Sca-1+/CD31− CSCs*^hTERT^* (Figure 4A–C). *MCP-1* siRNA-transfected Sca-1+/CD31− CSCs*^hTERT^* CM still showed significant reductions (78% in CoCl_2_-induced hypoxic injury model, and 85% in H_2_O_2_-induced oxidative stress model, respectively) in the proportions of apoptotic HL-1 cardiomyocytes compared to NC siRNA-transfected Sca-1+/CD31− CSCs*^hTERT^* CM (Figure 5D–G). Therefore, failure in an increase of the proportion of apoptotic HL-1 cardiomyocytes to the level of NC siRNA-transfected Sca-1+/CD31− CSCs*^hTERT^* CM could be due to partial silencing of *MCP-1* in Sca-1+/CD31− CSCs*^hTERT^*. Our findings are in line with previous studies that MCP-1 significantly decreased hypoxia-induced cell death in cardiomyocytes [36,37]. Martire *et al.* [38] demonstrated that cardiac overexpression of *MCP-1* in transgenic mice mimics ischemic preconditioning through stress-activated protein kinase (SAPK)/c-Jun N-terminal kinase (JNK) 1/2 activation, suggesting that a permanent activation of SAPK/JNK1/2 pathway in *MCP-1* transgenic mice could be involved in the development of cardiac resistance against ischemia. Furthermore, MCP-1 played a critical role in neuroprotection against in rat primary midbrain neurons [39], and protected the kidney during the acute inflammatory response following renal I/R injury [40]. Additional studies will be required to further elucidate precise mechanism(s) by which Sca-1+/CD31− CSCs*^hTERT^*-secreted MCP-1 leads to an increase in cardiomyocyte survival during hypoxia-induced injury.

In addition to MCP-1, we also found the dominant paracrine factors secreted by Sca-1+/CD31− CSCs*^hTERT^* are EGF, HGF R, IGF-1, IGF-2, IL-6, IL-1α, and TGF-β1. Previous studies also reported that anti-apoptotic roles of IGF-1 [41], HGF [42], TGF-β [21,43,44] promoted cardioprotection, resulting in promotion of myocardial repair. In the present study, we found that VEGF and IL-6 secreted from Sca-1+/CD31− CSCs*^hTERT^* CM were severely reduced compared to primary Sca-1+ CSCs CM. Recently, we also reported that secretion levels of IL-6 were severely reduced in ASCs*^TERT^* compared to that of primary ASCs [16]. VEGF levels were reduced in Sca-1+/CD34− ASCs*^TERT^* CM compared to primary ASCs CM, and Sca-1+/CD34+ ASCs*^TERT^* CM [16]. VEGF and IL-6 played critical roles in angiogenesis [45,46]. Primary Sca-1+ CSCs contained both mesenchymal stem-like CD31− and endothelial-like CD31+ subsets, whereas Sca-1+/CD31− CSCs*^hTERT^* were composed of homogeneous mesenchymal stem-like cells selected at the single cell level. Therefore, we speculate that the differences in VEGF and IL-6 levels secreted from Sca-1+/CD31− CSCs*^hTERT^* and primary Sca-1+ CSCs may reflect lineage-specific features between the two cell types. Further studies will be needed to elucidate the relationship between secretory profiles and functional differences in primary Sca-1+ CSCs, Sca-1+/CD31− CSCs*^hTERT^* and Sca-1+/CD31+ CSCs*^hTERT^*. Our findings and previous reports suggest that other paracrine factors secreted from Sca-1+/CD31− CSCs*^hTERT^*, besides MCP-1, could also contribute to a reduction in cardiomyocyte death during hypoxia-induced injury.

Interestingly, we also found that *MCP-1* silencing in Sca-1+/CD31− CSCs*^hTERT^* resulted in a significant reduction of IL-6 protein expression level. Morimoto *et al.* [47] revealed that a combination of IL-6 with MCP-1 synergistically stimulated and sustained STAT3 activation in cardiomyocytes. Furthermore, marked myocardial IL-6 secretion, STAT3 activation, and LV hypertrophy were observed after MI in transgenic mice overexpressing *MCP-1*, thereby resulting in the prevention of LV dysfunction and remodeling after MI [47]. Liu *et al.* [48] reported that anti-apoptotic effect of MCP-1 in fibroblasts was eliminated in the presence of anti-IL-6 neutralizing antibody. These results suggest that MCP-1-mediated pro-survival signaling was achieved via activation of IL-6 signaling.

## 4. Material and Methods

### 4.1. Isolation of Primary Sca-1+ CSCs

Animals were strictly treated in accordance with the Guidelines for Animal Care and Use of Korea University School of Medicine. All procedures were approved by the Korea University Institutional Ethics Committee for animal research (Seoul, Korea, 7 December 2009; KUIACUC-2011-208, Seoul, Korea, 31 May 2014; KUIACUC-2014-110). Heart tissues were dissected from six-week-old male ICR mice (Hanlim Experimental Animal Laboratory, Seoul, Korea). The mice were given an intraperitoneal (IP) injection anesthesia with a mixture of ketamine (44 mg/kg; Yuhan, Gunpo, Korea) and xylazine hydrochloride (0.75 mg/kg; Bayer AB, Leverkusen, Germany), and euthanized. Heart tissues were finely minced with scissors and washed with phosphate-buffered saline (PBS) three times at 4 °C. The heart tissues were digested with 0.1% collagenase type 2 (Worthington Biochemical Corporation, Lakewood, NJ, USA) for 30 min at 37 °C with shaking at 200 rpm, followed by filtration with a cell strainer (100 µm pore size; Falcon, Micronic, Lelystad, The Netherlands). The cell suspension was centrifuged at 680× *g* for 5 min and resuspended in PBS containing 3% fetal bovine serum (FBS; Invitrogen, Carlsbad, CA, USA). Enrichment of Sca-1+ CSCs was achieved by using MACS (Miltenyl Biotec, Sunnyvale, CA, USA). Briefly, the whole primary cell suspension was incubated with phycoerythrin (PE)-conjugated anti-Sca-1 antibody (BD Biosciences, Franklin Lakes, NJ, USA) for 15 min at 4 °C, washed in MACS buffer (PBS pH 7.4, 0.5% bovine serum albumin (BSA) and 2 mM ethylenediaminetetraacetic acid), incubated with anti-PE micro beads (Miltenyl Biotec) for 15 min at 4 °C, and washed with MACS buffer. Samples were passed through a MACS column set up in a Miltenyl magnet, and the Sca-1+ CSCs were eluted from the column by washing with MACS buffer. To increase the purity of Sca-1+ CSCs, MACS was performed three more times. The purity of Sca-1+ CSCs was analyzed by flow cytometry (Becton Dickinson Systems, San Jose, CA, USA).

### 4.2. Construction of a Retroviral Vector Encoding hTERT-GFP and Production of Retroviruses

We generated the pLPCX-*hTERT*-IRES-*GFP* vector, a *Bgl*II-*Sal*I fragment containing *hTERT* cDNA was amplified by PCR using pCI-neo-hEST2 (Addgene, Cambridge, MA, USA) as a template. The *Bgl*II-*Sal*I digested PCR fragment containing *hTERT* cDNA was inserted into the *Bgl*II-*Sal*I site of pIRES2-*GFP* vector (BD Biosciences). Finally, the *Bgl*II-*Cla*I digested PCR fragment containing *hTERT*-IRES-*GFP* cDNA was finally inserted into the *Bgl*II-*Cla*I site of the pLPCX vector (BD Biosciences). The detailed cloning strategy of a retroviral vector encoding *hTERT*-IRES-*GFP* and production of retroviruses expressing pLPCX-*hTERT*-IRES-*GFP* in 293GPG packaging cell line [49] was performed as previously described [16].

### 4.3. Generation of Immortalized Sca-1+ CSCs

MACS-sorted Sca-1+ CSCs were plated at 2 × 10^5^ cells in 6-cm culture dishes in Dulbecco’s Modified Eagle’s Medium (DMEM)-low glucose (LG) supplemented with 10% FBS and 100 U/mL penicillin/streptomycin (P/S). Cells were infected with retroviruses harboring pLPCX-*hTERT*-IRES-*GFP* at 60% confluence for three days. The cells were selected in medium against 0.5 µg/mL puromycin by repeated sub-culturing at a 1:3 ratio three times per week during a three-week subculture period in 10-cm culture dishes. For clonal analysis, the selected cells were plated in 96-well plates at one cell per 100 µL by limiting dilution in DMEM-LG supplemented with 10% FBS and 100 U/mL P/S, as described previously [6]. Briefly, wells containing one cell per well were only selected by visual inspection 24 h after plating, and were further cultured for 12 days. Among 20 clones derived from a single cell, two clones were finally selected based on microscopic examination of morphology, proliferation, GFP expression, and *hTERT* expression. 

### 4.4. Phenotypic Characterization of Sca-1+ CSCs by Immunostaining

Primary CSCs and *hTERT*-immortalized Sca-1+ CSC lines were plated onto coverslips coated with 0.1% (*w*/*v*) gelatin in 24-well plates. The cells were fixed with 4% paraformaldehyde in PBS for 10 min and washed with PBS + 0.1% Tween-20 (PBST). Cells were blocked for nonspecific binding by incubation in 5% normal goat serum (NGS; Invitrogen) in PBST for 30 min. Next, cells were stained for 30 min with the following primary antibodies: CD14, CD29, CD31, CD44, CD45, CD71, CD90, CD106, CD117, and Sca-1 (all from BD Biosciences), CD34 (e-Bioscience, San Diego, CA, USA), and CD133 (e-Bioscience). Cells were stained with Alexa Fluor 594-conjugated secondary antibodies (Molecular Probes, Eugene, OR, USA) for 30 min and washed three times in PBST. For control experiments, cells were stained with secondary antibodies only. Nuclei were stained with 4′,6-diamidino-2-phenylindole dihydrochloride (DAPI; Sigma-Aldrich, St. Louis, MO, USA), and cells were mounted using fluorescent mounting medium (DAKO, Glostrup, Denmark). Fluorescence images were obtained using a TEFM Epi-fluorescence system attached to an inverted microscope (Olympus, Tokyo, Japan) or were acquired with a confocal fluorescence microscope (LSM710, Carl Zeiss, Oberkochen, Germany).

### 4.5. Phenotypic Characterization of Sca-1+ CSCs by Flow Cytometry

Primary CSCs and *hTERT*-immortalized Sca-1+ CSC lines were fixed with 4% paraformaldehyde in PBS for 10 min at room temperature. The cells were subsequently incubated for 20 min at 4 °C with the following primary antibodies: CD14, CD29, CD31, CD34, CD44, CD45, CD71, CD90, CD106, CD117, CD133, and Sca-1. After washing twice with PBS + 2% FBS, cells were incubated with fluorescein isothiocyanate (FITC)-conjugated goat anti-rat antibodies (e-Bioscience) for 15 min at 4 °C. For control experiments, the cells were stained with secondary antibodies only. After washing twice with PBS + 2% FBS, thirty thousand cells for each sample were analyzed on a FACSCalibur flow cytometer (BD Biosciences). Data were analyzed using CellQuest Pro software (BD Biosciences).

### 4.6. Differentiation Potential of Sca-1+ CSCs

Primary CSCs and Sca-1+/CD31− CSCs*^hTERT^* were plated at a density of 1~2 × 10^4^ cells/mL in 24-well plates containing 0.1% (*w*/*v*) gelatin-coated glass coverslips. Cells were cultured in DMEM-LG supplemented with 10% FBS and 100 U/mL P/S for 2–3 days. Cardiomyogenic differentiation of primary CSCs and Sca-1+/CD31− CSCs*^hTERT^* was induced by incubation in DMEM-LG supplemented with 10% FBS, 100 U/mL P/S, and 1 µM 5-azacytidine (Sigma-Aldrich) for 21 days. Cultures were maintained by media exchange every 3~4 days. Endothelial differentiation of primary CSCs and Sca-1+/CD31− CSCs*^hTERT^* was induced by incubation in 60% DMEM-LG and 40% MCDB-201 (Sigma-Aldrich), supplemented with 1× insulin-transferrin-selenium, 1× linoleic acid-BSA, 10^−8^ M dexamethasone, 10^−4^ M ascorbic acid 2-phosphate (all from Sigma-Aldrich), and 100 U/mL P/S plus 20 ng/mL VEGF (R and D Systems, Minneapolis, MN, USA) for 21 days. Cultures were maintained by media exchange every 3–4 days. To assess cardiac or endothelial differentiation, the cells were fixed with 4% paraformaldehyde in PBS for 10 min, washed with PBST, and permeabilized with 0.1% Triton X-100 in PBS for 30 min. Cells were washed with PBST and blocked for nonspecific binding by incubation in 5% NGS in PBST for 30 min. Then, the cells were incubated overnight at 4 °C with the following primary antibodies: anti-MLC (Sigma-Aldrich), anti-cTnI (Abcam, Cambridge, UK), anti-cardiac troponin T (cTnT; Developmental Studies Hybridoma Bank, Iowa City, IA, USA), and anti-vWF (DAKO). After washing three times with PBST, the cells were stained with Alexa Fluor 488- or 594-conjugated secondary antibodies (all from Molecular Probes) for 30 min, and washed three times in PBST. For control experiments, the cells were stained with secondary antibodies only. Nuclei were stained with DAPI (Sigma-Aldrich). The cells were mounted with fluorescent mounting medium (DAKO). Fluorescence images were obtained with a TE-FM Epi-Fluorescence system attached to an inverted microscope (Olympus). Adipogenic differentiation of primary CSCs and Sca-1+/CD31− CSCs*^hTERT^* was induced by incubation in DMEM-LG supplemented with 5% FBS and 100 U/mL P/S, 1 μM dexamethasone, 10 μg/mL insulin, 100 μM indomethacin, and 0.5 μM methyl-isobutylxanthin (all from Sigma-Aldrich) for 10 days. Culture media were changed every three days. Adipogenic differentiation was assessed on day 10 using Oil Red O (Sigma-Aldrich) stain as an indicator of intracellular lipid accumulation. The cells were fixed with 4% paraformaldehyde in PBS for 20 min, washed with 60% isopropanol, and stained with 0.3% Oil Red O solution in 60% isopropanol for 10 min. After washing three times with water, cells were de-stained in 100% isopropanol for 15 min. Osteogenic differentiation of primary CSCs and Sca-1+/CD31− CSCs*^hTERT^* was induced by incubation in culture medium with 1 μM dexamethasone, 10 mM glycerophosphate, and 50 μM ascorbic acid (all from Sigma-Aldrich) for 21 days. Osteogenic differentiation was determined by Alizarin Red S (Sigma-Aldrich) staining.

### 4.7. Real-Time PCR

Total RNAs were extracted from primary CSCs, Sca-1+/CD31− CSCs*^hTERT^*, *MCP-1* siRNA-transfected Sca-1+/CD31− CSCs*^hTERT^* or NC siRNA-transfected Sca-1+/CD31− CSCs*^hTERT^* using Trizol reagent (Invitrogen). The concentrations of total RNAs were determined using a Nanodrop 1000 spectrophotometer (Thermo Scientific, Waltham, MA, USA). First-strand cDNA was synthesized from 0.5 μg of DNase-treated total RNA using 0.5 μg of random hexamers (Invitrogen), and 200 U of Moloney murine leukemia virus reverse transcriptase (Invitrogen) at 37 °C for 60 min in a 20 μL volume. Real-time PCR was performed using a real-time PCR thermal cycler (Bio-Rad Laboratories, Hercules, CA, USA). Each reaction contained 12.5 μL 2X SYBR Green PCR Mix (Bio-Rad Laboratories), 1.5 μL forward primer (5 μM), 1.5 μL reverse primer (5 μM), 5 μL of a 1:10 dilution of cDNA, and 4.5 μL H_2_O. The primers used for real-time PCR were as follows: 5′-TGAAAGTAGAGGATTGCCACTG-3′, 5′-AGCCAGAACAGGAACGTAGC-3′ (*mTERT*, 109 bp); 5′-CGGAAGAGTGTCTGGAGCAA-3′, 5′-GGATGAAGCGGAGTCTGGA-3′ (*hTERT*, 145 bp); 5′-GGTGATCGCAACCCTAGC-3′, 5′-TGTGTCGGCTGGATAGGC-3′ (*mMCP-1*, 111 bp); 5′-TTCACCACCATGGAGAAGGC-3′, 5′-GGCATGGACTGTGGTCATGA-3′ (*mGAPDH*, 236 bp). Real-time PCR data were pooled from three independent experiments. Relative gene expression levels were quantified based on Ct and normalized to the reference gene, GAPDH.

### 4.8. WST-1 Proliferation Assay

Sca-1+/CD31− CSCs*^hTERT^* were plated at a density of 1 × 10^3^ cells/well in a 96-well plate. Cells were cultured in Mesencult mesenchymal stem cell (MSC) Basal Medium supplemented with 10% Mesencult MSC Stimulatory Supplements (StemCell Technologies Inc., Vancouver, BC, Canada) and 100 U/mL P/S. The cells were analyzed at days 1, 2, 3, 4, 5, and 6 using a WST-1 assay (Roche Applied Science, Mannheim, Germany). In brief, the cells were incubated at a concentration of 10 μM of WST-1 for 2 h. The cells were then incubated until color development was sufficient for photometric detection. The reaction product was quantified by measuring absorbance using an ELISA reader (Molecular Devices, Sunnyvale, CA, USA) at 440 and 690 nm. Data were analyzed using SoftMax^®^ Pro quantification of absorbance analysis software (Molecular Devices).

### 4.9. Antibody Array Detection of Sca-1+ CSCs CM

Primary CSCs and Sca-1+/CD31− CSCs*^hTERT^* were seeded at 1 × 10^5^ cells in 10-cm tissue culture dishes and allowed to reach ~80% confluence in Mesencult Complete Medium. The medium was then changed to Mesencult MSC Basal Medium supplemented with 2% FBS and 100 U/mL P/S, and further cultured for 24 h. Primary CSCs CM, Sca-1+/CD31− CSCs*^hTERT^* lysate and CM were collected. Mouse cytokine antibody arrays (RayBiotech, Norcross, GA, USA) that include in duplicate 21 cytokines and an appropriate positive and negative control were customized. The analyzed proteins included: EGF, TGF-β1, HGF, IGF-1, IGF-2, MCP-1, VEGF, stromal cell-derived factor 1 (SDF-1), bFGF, E-Cadherin, HGF R, IFN-γ, IL-10, TNF-α, IL-6, IL-1α, IL-1β, IL-1ra, Leptin, CT-1, and MCP-5. Densitometric quantification of blotting spots was performed using Quantity One software (Bio-Rad).

### 4.10. Apoptosis Assay of HL-1 Cardiomyocytes

Sca-1+/CD31− CSCs*^hTERT^* were cultured on six-well plates at a density of 5 × 10^4^ cells/well and transfected with 50 nM of *MCP-1* siRNA duplexes (5′-CACAACCACCTCAAGCACT-3′) or NC siRNA (all from Bioneer) using Lipofectamine RNAiMAX (Invitrogen) for 48 h as suggested by the manufacturer. For CoCl_2_- (Sigma-Aldrich) or H_2_O_2_-induced hypoxia, HL-1 cells, a cardiomyocyte cell line that continuously divides and spontaneously contracts while maintaining a differentiated cardiac phenotype [50] was used. HL-1 cardiomyocytes were seeded at 6 × 10^5^ cells in fibronectin (12.5 mg/L)–gelatin (0.02%) coated six-well culture dishes and allowed to reach ~80% confluence in Claycomb medium (Sigma-Aldrich) supplemented with 10% FBS (Invitrogen), 2 mM l-glutamine, 0.1 mM norepinephrine, 0.3 mM ascorbic acid, and 100 U/mL P/S in a humidified 37 °C/5% CO_2_ incubator. The cells then were treated with 150 µM CoCl_2_ or 200 µM H_2_O_2_ for 24 h in Mesencult MSC Basal Medium supplemented with 2% FBS and 100 U/mL P/S in the presence of *MCP-1* siRNA-transfected Sca-1+/CD31− CSCs*^hTERT^* CM or NC siRNA-transfected Sca-1+/CD31− CSCs*^hTERT^* CM. Annexin V (AV) and propidium iodide (PI) staining were performed using a FITC Annexin V Apoptosis Detection Kit II (BD Biosciences) according to the manufacturer's instructions, and then flow cytometric analysis was performed. Viable cells were counted using a hemocytometer after staining with 0.2% trypan blue (Invitrogen) to reveal the dead cells.

### 4.11. Statistical Analysis

All statistical values are expressed as the mean ± standard deviation (SD). Significant differences between means were determined using the Student’s *t*-test or by analysis of variance followed by the Student–Newman–Keuls test. Statistical significance was set at *p* < 0.05.

## 5. Conclusions

In the present study, we established that Sca-1+/CD31− CSCs*^hTERT^* exhibits long-term proliferation capacity and multi-differentiation potential similar to those of primary Sca-1+ CSCs. We found that Sca-1+/CD31− CSCs*^hTERT^* dominantly secrete EGF, TGF-β1, IGF-1, IGF-2, MCP-1, HGF R, and IL-6. Among these, MCP-1 was the most predominant factor in Sca-1+/CD31− CSCs*^hTERT^* CM. Sca-1+/CD31− CSCs*^hTERT^* CM protected HL-1 cardiomyocytes from hypoxic injury partly via a MCP-1-dependent mechanism. Thus, Sca-1+/CD31− CSCs*^hTERT^* are valuable sources for *in vitro* and *in vivo* studies in the cardiovascular field.

## Supplementary Materials

Supplementary materials can be found at http://www.mdpi.com/1422-0067/17/6/800/s1.
