# Supplementary material for: Cardiac Stem Cell Secretome Protects Cardiomyocytes from Hypoxic Injury Partly via Monocyte Chemotactic Protein-1-Dependent Mechanism
Source: Int J Mol Sci. 2016 May 24;17(6):800. doi: 10.3390/ijms17060800 (PMC4926334; doi:10.3390/ijms17060800)
Supplement: Supplementary file 1 [file ijms-17-00800-s001.pdf]

# Supplementary Materials: Cardiac Stem Cell Secretome Protects Cardiomyocytes from Hypoxic Injury Partly via Monocyte Chemotactic Protein-1-Dependent Mechanism

**Chi-Yeon Park, Seung-Cheol Choi, Jong-Ho Kim, Ji-Hyun Choi, Hyung Joon Joo, Soon Jun Hong and Do-Sun Lim**

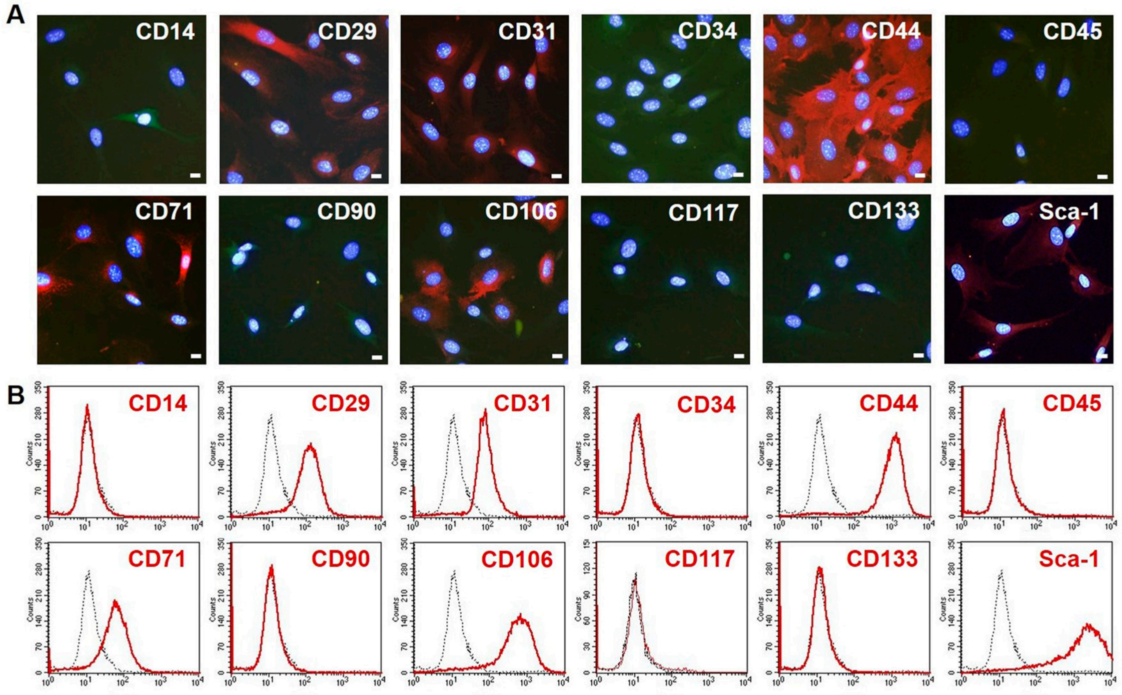


**Figure S1.** Phenotypic characterization of Sca-1+/CD31+ CSCs$^{hTERT}$. Sca-1+/CD31+ CSCs$^{hTERT}$ were analyzed by immunostaining (**A**) and flow cytometry (**B**) with different cell surface antibodies. Scale bars = 20 μm.

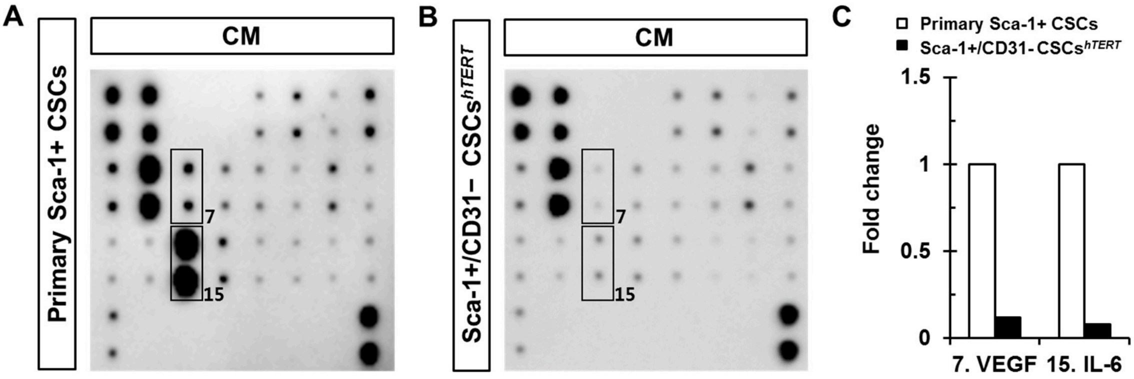


**Figure S2.** Comparison of paracrine factors secreted from primary Sca-1+ CSCs and Sca-1+/CD31− CSCs$^{hTERT}$. (**A**) CMs secreted from primary Sca-1+ CSCs and Sca-1+/CD31− CSCs$^{hTERT}$ were subjected to a mouse cytokine antibody array detecting 21 cytokines in duplicate. Solid lined boxes indicate differentially secreted paracrine factors, VEGF (marked by number 7) and IL-6 (marked by number 15) between primary Sca-1+ CSCs (**A**) and Sca-1+/CD31− CSCs$^{hTERT}$ (**B**); (**C**) VEGF and IL-6 secreted from primary Sca-1+ CSCs and Sca-1+/CD31− CSCs$^{hTERT}$ were subjected to densitometry and are presented as fold changes for individual cytokines, taking primary Sca-1+ CSCs as a one-fold value.

**Table S1.** Selection of *hTERT*-immortalized Sca-1+ CSC lines by limiting dilution.

| **Clone Number** | **Cell Number** | | | | | |
|---|---|---|---|---|---|---|
| | **Day 1** | **Day 5** | **Day 6** | **Day 7** | **Day 8** | **Day 12** |
| 1 | 1 | 2 | 3 | – | – | – |
| 2 | 1 | 5 | 6 | 14 | 16 | 17 |
| 3 | 1 | 6 | 11 | 24 | 44 | >200 |
| 4 | 1 | 2 | 1 | 1 | – | – |
| 5 | 1 | 2 | 3 | 8 | 9 | – |
| 6 | 1 | 1 | 1 | – | – | – |
| 7 | 1 | 1 | 2 | 4 | 1 | – |
| 8 | 1 | 11 | 19 | 30 | 51 | >200 |
| 9 | 1 | 2 | 1 | 1 | 1 | – |
| 10 | 1 | 2 | 2 | 2 | 2 | 3 |
| 11 | 1 | 7 | 14 | 19 | 38 | 105 |
| 12 | 1 | 4 | 2 | – | – | – |
| 13 | 1 | 5 | 10 | 32 | 50 | 29 |
| 14 | 1 | 4 | 3 | 3 | – | – |
| 15 | 1 | 3 | 9 | 4 | 2 | – |
| 16 | 1 | 9 | 13 | 19 | 30 | 89 |
| 17 | 1 | 10 | 23 | 29 | 88 | >200 |
| 18 | 1 | 6 | 21 | 15 | 16 | – |
| 19 | 1 | – | – | – | – | – |
| 20 | 1 | 3 | 8 | 10 | 9 | 7 |
